# Unbiased autoantibody screening using nucleic acid protein programmable array in pediatric autoimmune neuropsychiatric disorder associated with streptococcal infections

**DOI:** 10.3389/fnbeh.2026.1774848

**Published:** 2026-04-29

**Authors:** Jelena Eremija, Michael Daines, Sydney Rice, Juliette C. Madan, Susan Swedo, Fayez K. Ghishan, Pawel R. Kiela

**Affiliations:** 1 Department of Pediatrics, The Children’s Postinfectious Autoimmune Encephalopathy (CPAE) Center of Excellence, Daniel Cracchiolo Institute for Pediatric Autoimmune Disease Research, Steele Children's Research Center, University of Arizona Health Sciences Center, Tucson, AZ, United States; 2 Division of Child Psychiatry, Neuroimmune Psychiatric Disorders Program, Departments of Psychiatry and Pediatrics, Dartmouth Hitchcock Medical Center, Lebanon, NH, United States; 3 Department of Epidemiology, Geisel School of Medicine at Dartmouth, Hanover, NH, United States; 4 National Institute of Mental Health, National Institutes of Health, Bethesda, MD, United States; 5 Department of Immunobiology, University of Arizona Health Sciences Center, Tucson, AZ, United States

**Keywords:** autoantigen, biomarker, children, IgG, NAPPA array, serum

## Abstract

**Introduction:**

Pediatric autoimmune neuropsychiatric disorder associated with streptococcal infections (PANDAS) is characterized by the abrupt onset of obsessive-compulsive disorder and/or tics, along with other neuropsychiatric symptoms in children, in the setting of Group A beta-hemolytic streptococcus infection and presumed immune dysregulation. Current diagnostic testing has limited utility in diagnosis of PANDAS.

**Methods:**

The Nucleic Acid Protein Programmable Array (NAPPA) is a protein expression system that enables the study of protein-autoantibody interactions. We employed this system for unbiased screening of potential autoimmune targets in PANDAS.

**Results:**

Initially, we compared children with PANDAS to patients with autism spectrum disorder (ASD) and identified 1,235 non-overlapping protein targets. These targets were subsequently used to re-screen samples from PANDAS, ASD and healthy controls (HC). In the secondary screen, 117 autoantibody targets were exclusively detected in PANDAS and absent from both ASD and HC groups. However, autoantibody profiles were highly heterogeneous: 67.5% of PANDAS-positive targets were reactive in only a single patient, and no individual protein or multi-protein panel classifier achieved reliable diagnostic discrimination under proper cross-validation. Targets that discriminated PANDAS from HC largely failed to discriminate PANDAS from ASD, and vice versa, indicating that autoantibody-based diagnosis is confounded by reactivity shared across neuropsychiatric conditions. Despite poor diagnostic performance, functional enrichment analysis revealed that PANDAS-specific targets were significantly overrepresented among transcription factors, apoptosis regulators, chromatin/epigenetic modifiers, neural development proteins, and immune-regulatory molecules, with the majority being intracellular proteins enriched for intrinsically disordered regions.

**Discussion:**

These findings support the hypothesis of autoimmunity and epitope spreading as features of PANDAS but indicate that the heterogeneity of the humoral response represents a fundamental challenge for serological diagnosis. Future studies with larger cohorts and complementary approaches are necessary to determine whether autoantibody signatures can be leveraged for clinical utility.

## Introduction

Although the etiology of neuropsychiatric disorders remains incompletely understood, research has yielded data emphasizing the role of autoimmunity and immune dysregulation in an increasing number of these illnesses (Benros et al., 2011; Cullen et al., 2019; Marrie et al., 2018). This evolving understanding of a complex interplay between immunity and the nervous system has led to the development of immunoneuropsychiatry—a novel approach to the study, assessment, and treatment of neuropsychiatric disorders (Pape et al., 2019). An example of neurological and psychiatric/behavioral deficits developing in the context of presumed immune dysregulation is Pediatric Autoimmune Neuropsychiatric Disorder associated with Streptococcal infections (PANDAS). PANDAS was originally described in the 1990s (Swedo et al., 1998) and was defined by the obsessive-compulsive disorder (OCD) and/or tic disorder, prepubertal onset, episodic symptom course, association with Group A beta-hemolytic streptococcal (GABHS) infection, and accompanying neurological abnormalities. Over time, with more research and focus on this disorder, a broadened definition was developed in order to encompass a wider spectrum of neuropsychiatric conditions: “Pediatric Acute-onset Neuropsychiatric Syndrome” or PANS (Swedo et al., 2012), which is now considered an umbrella condition that also encompasses PANDAS, as recently described in the American Academy of Pediatrics clinical report (AAP Board of Directors, 2025). PANDAS is a rare condition, with a retrospective study across three U. S. academic centers estimating its annual incidence at approximately 1 in 11,765 children aged 3–12 years (Wald et al., 2023).

Although the pathophysiology of PANDAS remains incompletely elucidated, there is increasing evidence, particularly in the past decade, to suggest that immune dysregulation plays a key role (Cutforth et al., 2016; Melamed et al., 2021; Murphy et al., 2015; Shimasaki et al., 2020). The etiopathogenesis is hypothesized to be similar to Sydenham chorea (SC), a well-characterized post-streptococcal autoimmune condition in which Group A streptococcal infections provoke the creation of cross-reactive anti-neuronal antibodies that induce neuroinflammation, particularly within the basal ganglia (Chain et al., 2020; Cunningham, 2012; Cunningham, 2014; Swedo et al., 2022). Indeed, IgG antibodies from children with PANDAS have been shown to bind to a variety of antigens, such as cholinergic interneurons (CINs) in the striatum (Xu et al., 2021; Kirvan et al., 2006). Candidate autoantibody targets shared between SC and PANDAS were used to develop the Cunningham Panel (Chain et al., 2020). This panel has also been used as a tool to make therapeutic decisions in some children with autism (Connery et al., 2018). The Cunningham Panel (currently marketed as the Autoimmune Brain Panel, Moleculera Biosciences; Oklahoma City, OK) includes five assays. Four assays measure antibodies targeting dopamine D1 receptor (D1R), dopamine D2L receptor (D2LR), lysoganglioside-GM1, and tubulin. The fifth is a cell stimulation assay evaluating serum-induced activation of calcium/calmodulin-dependent protein kinase II (CaMKII) activity in the human neuroblastoma cell line. The Autoimmune Brain Panel has been proven to have some utility in PANDAS (Shimasaki et al., 2020; Chain et al., 2020), although several studies questioned its reliability and correlation with disease activity (Morris-Berry et al., 2013; Bejerot and Hesselmark, 2019; Hesselmark and Bejerot, 2017a; Hesselmark and Bejerot, 2017b). A randomized controlled trial found that only ANA and CaMKII positivity predicted treatment response to both intravenous immunoglobulin (IVIG) and placebo, while other components had no predictive value (Williams et al., 2016).

PANS/PANDAS diagnosis remains challenging due to the complex presentation, relapsing–remitting course, and not yet fully understood etiology of the disorder. In 2015, guidelines for assessment were published (Chang et al., 2015), but they were based on clinical features only and thus lacked precision and inter-rater reliability. To address the critical need for specific, precise, objective, and reproducible tests to aid in diagnosis, we sought to identify novel candidate autoantigens that might be used with reliability and validity not only for diagnostic evaluations but also as an aid in predicting disease activity and treatment response. We employed an unbiased autoantibody screening using Nucleic Acid Protein Programmable Array (NAPPA; ASU Biodesign Institute), a high-throughput, fast, and reliable *in vitro* cell-free full-length protein expression system (Song et al., 2021; Bian et al., 2017). We observed a high heterogeneity in autoantibody targets among PANDAS subjects, which pointed to potential difficulties in utilizing small subsets of targets for diagnostic purposes with high sensitivity. Our data also provide identification of putative targets with a potential role in disease pathogenesis, albeit also with a considerable heterogeneity among subjects.

## Materials and methods

### Ethics statement

Deidentified serum samples from pediatric subjects with symptomatic (active) PANDAS, autism spectrum disorder (ASD, as non-neurotypical controls), and healthy age-matched subjects were obtained from Dr. Susan Swedo at the National Institute of Mental Health (NIMH) and from the Children’s Postinfectious Autoimmune Encephalopathy (CPAE) clinic at the University of Arizona (UA). NIMH samples were collected under approved IRB protocols 09-M-N216 and 11-M-0058 (Material Transfer Agreement NIMH Ref. #2018-0220). UA samples were obtained under an approved IRB protocol # 1707640615. Informed consents/assents were obtained from subjects and their parents/guardians at the time of enrolment. Additional deidentified serum samples from age and sex-matched controls were obtained from US Biolab Corporation, Inc. (Rockville, MD, USA).

### Participants

A total of 42 participants were classified into three groups: PANDAS (*n* = 13), ASD (*n* = 9), and healthy controls (HC; *n* = 20). The average age in the PANDAS group was 8.6 ± 3.25 years, 10.6 ± 2.7 years in HC, and 6.5 ± 3.28 years in the ASD group (Table 1). The PANDAS subjects were diagnosed at the NIMH or UA CPAE clinic and met the diagnostic criteria for PANDAS, which included sudden onset or exacerbation of OCD and/or tic disorders following a streptococcal infection as well as associated secondary characteristics (Swedo et al., 1998). The period between the onset of symptoms and serum sample collection ranged from 0.12–2.29 years (median duration/time 0.79 years). All PANDAS subjects were symptomatic, and only one received IVIG therapy at the time of sample collection (#PS31). The HC group had no history of neuropsychiatric or autoimmune disorders. The autism group met the ASD DSM-5 diagnostic criteria (American Psychiatric Association, 2013). The selection of ASD as a neuropsychiatric comparator was driven by several factors. First, ASD and PANDAS exhibit significant phenotypic overlap, such as repetitive behaviors, anxiety, and social withdrawal. These overlapping symptoms can pose challenges in clinical practice when differentiating between the two conditions. Second, both conditions have been linked to immune dysregulation and neuroinflammation. This includes elevated pro-inflammatory cytokines, altered T cell profiles, and, in the case of ASD, reports of circulating autoantibodies directed against brain antigens such as anti-neuronal and anti-nuclear antibodies. This shared immunological background makes ASD an informative comparator for assessing whether PANDAS-associated autoantibodies represent disease-specific autoimmunity or broader neuroimmune activation common to various neuropsychiatric conditions. Third, from a practical diagnostic perspective, distinguishing PANDAS from other neurodevelopmental and neuropsychiatric disorders presenting with overlapping symptoms is a crucial clinical challenge. Comparing PANDAS to healthy controls alone would overestimate the diagnostic specificity of any candidate biomarker. Therefore, the inclusion of ASD serves both a biological purpose (testing the specificity of autoantibody targets against a condition with known immune involvement) and a clinical one (evaluating its discriminatory performance in a realistic differential diagnosis scenario).

**Table 1:** Patient demographics.

Age and sex of participants	HC	PANDAS	ASD
Age [years; mean (SD)]	10.6 (2.68)	8.6 (3.25)	6.5 (3.28)*
Males, *n* (%)	10 (50%)	3 (23%)	5 (56%)
Females, *n* (%)	10 (50%)	10 (77%)	4 (44%)

*ANOVA *p* = 0.0053, asterisk indicates a statistical difference in the age of subjects with ASD compared to healthy controls (HC; Tukey test *p* = 0.0043).

### Neurofilament light chain (NfL) assay

NfL is a non-specific biomarker that reflects neuroaxonal injury/degeneration in various psychiatric and neurodegenerative disorders (Bavato et al., 2024; Ghezzi and Neuteboom, 2024), as it is released into the blood when neuronal cells are damaged (Gaetani et al., 2019). Nine serum samples from the HC group, 9 ASD, and 20 PANDAS (such as 7 additional subjects enrolled after the NAPPA array analysis was complete) were analyzed for the NfL concentration using the Simoa™ NF-Light® kit (Quanterix Cat #103186) by PBL Assay Science (Piscataway, NJ, USA). Calibrators were run in neat (undiluted) serum in duplicate. Samples were diluted offline at 1:6 and were further diluted at 1:4 onboard for a final dilution factor of 1:24 and processed in duplicate. Two controls provided in the kit were processed at a 1:4 onboard dilution in duplicate. Samples were analyzed using the HD-X Analyzer software package (Quanterix), which uses proprietary curve fit weighing algorithms to generate a four-parameter log calibration curve from the “average enzymes per bead” AEB values obtained from each respective NfL calibrator. These calibration curves were then used to back-interpolate the samples for NfL levels. The concentrations are reported in picogram/mL (pg/mL), and sample values are corrected for the dilution factor.

### Nucleic acid protein programmable arrays (NAPPA)

**Figure 1:**
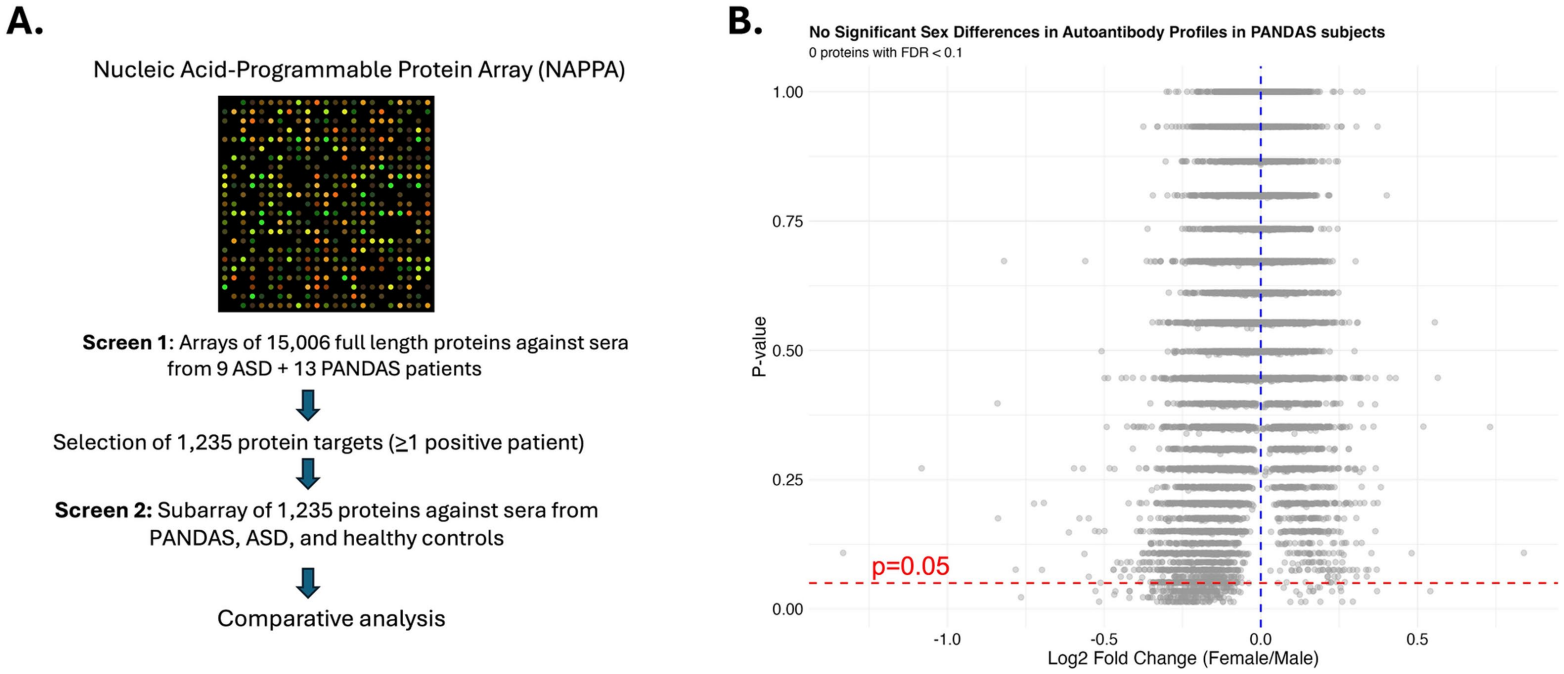
**(A)** Overview diagram of the approach and sample analysis pipeline. **(B)** Sex did not affect autoreactivity in PANDAS subjects. Volcano plot with the results of Mann–Whitney U test with FDR multiple testing correction were used to test whether sex affected the normalized intensity of any protein target among PANDAS subjects in our initial screen of 15,006 full-length proteins. None of the targets reached a significant difference between male and female PANDAS subjects at P_adj_ < 0.1.

NAPPA arrays were manufactured by the NAPPA Protein Array Facility at the Arizona State University, the Virginia G. Piper Biodesign Center for Personalized Diagnostics, as previously described (Song et al., 2021; Takulapalli et al., 2012; Song et al., 2017). Briefly, 15,006 full-length ORF clones having the genes of interest with a C-terminal GST tag were obtained from the DNASU Plasmid Repository.^1^ Plasmid DNA was purified using a mini-prep kit (Macherey-Nagel), and DNA concentrations were normalized to 100 ng/μL. Silicon nanowell substrates were coated with (3-aminopropyl) triethoxysilane (Thermo Scientific), and at the time of screening, the plasmid DNA was printed using a piezo electric printer. Proteins were expressed from plasmid DNA using an *in vitro* transcription and translation kit (Thermo Scientific). The printing quality of a batch was determined by expressing a random slide from the batch with the IVTT kit, followed by the detection of GST-tagged proteins with Mouse anti-GST antibody (Cell Signaling, #2624S) and Alexa 555 Goat anti-mouse IgG antibody (Invitrogen, #A-21422). The NAPPA platform also included positive and negative controls, such as GST, BSA, and empty plasmids. Proteins were produced *in situ* using the IVTT kit and displayed on NAPPA arrays. Patient serum samples were diluted at 1:200 in PBST with 5% milk and added to the microarrays, followed by overnight rocking at 4 °C. After washing with PBST, human IgG autoantibodies were detected by 1:3,000 diluted biotinylated anti-human IgG (Invitrogen), followed by Alexa 555-conjugated streptavidin (Invitrogen). Scanned microarray images were analyzed by the ArrayPro image analysis software. Antibody reactivity of each spot was normalized by division by the median spot intensity of each corresponding microarray. This normalized intensity value is denoted as median normalized intensity (MNI). A positive “hit” was defined as MNI > 2 (twofold over the median fluorescence intensity per the entire respective array). Initial screening with 15,006 full-length proteins was conducted with samples from ASD and PANDAS subjects only to capture potential PANDAS-specific targets and to reduce the cost of screening. This screening yielded 1,235 potential “hits” with at least one of the PANDAS or ASD samples showing MNI > 2. The 1,235 ORF clones were then re-arrayed for a secondary screen with samples from HC, PANDAS, and ASD subjects (Figure 1A). The same quantitative approach was applied at the second round of screening.

### ROC analysis of individual autoantibody targets

To evaluate the diagnostic performance of individual autoantibody targets for discriminating PANDAS from healthy controls (HC), receiver operating characteristic (ROC) analysis was performed for each of the 1,235 protein targets in the secondary screen. For each protein, the continuous median normalized intensity (MNI) value served as the diagnostic test variable, with PANDAS status as the binary outcome. The area under the ROC curve (AUC) was computed using the non-parametric Mann–Whitney U statistic, which is mathematically equivalent to the AUC and represents the probability that a randomly selected PANDAS subject has a higher MNI value than a randomly selected HC subject. Statistical significance of individual AUC values was assessed using the one-sided Mann–Whitney U test, with adjustment for multiple comparisons by the Benjamini–Hochberg false discovery rate (FDR) procedure.

### Panel-based diagnostic performance evaluation

Given the high heterogeneity of autoantibody profiles observed across PANDAS subjects, multi-protein panel classifiers were evaluated. Autoantibody reactivity data were binarized using the MNI > 2 threshold. L2-regularized logistic regression classifiers were trained using panels of *k* = 5, 10, 20, and 50 features selected by the chi-squared (*χ*^2^) test. To avoid circular analysis (where features are selected on the same data used for evaluation), a nested leave-one-out cross-validation (LOOCV) strategy was employed: within each LOOCV fold, feature selection was performed exclusively on the training samples (*n* − 1), and the held-out sample was classified using only features selected from training data. The predicted probability of PANDAS from each left-out sample was collected across all folds to construct ROC curves and compute AUC values. This nested design ensures that reported AUCs reflect the true out-of-sample generalizability of the panel approach and are not inflated by data leakage.

### Assessment of autoantibody profile heterogeneity

The degree of autoantibody target sharing among PANDAS subjects was quantified by computing, for each protein with at least one positive PANDAS hit (MNI > 2), and the number of PANDAS subjects showing positivity. Overlap of positive targets across the three diagnostic groups (PANDAS, ASD, HC) was enumerated. Per-subject autoantibody burden was assessed as the total number of positive targets (MNI > 2) per individual. All statistical analyses were performed using Python 3 with scikit-learn (v1.3), SciPy (v1.11), and statsmodels (v0.14).

### Three-group diagnostic performance evaluation

To assess whether autoantibody targets identified as discriminators of PANDAS from healthy controls (HC) also distinguish PANDAS from autism spectrum disorder (ASD), ROC analysis was performed for three pairwise comparisons: PANDAS versus HC (*n* = 13 vs. 20), PANDAS versus ASD (*n* = 13 vs. 9), and PANDAS versus all non-PANDAS subjects (ASD + HC combined; *n* = 13 vs. 29). For each of the 1,235 protein targets, the AUC was computed independently for each comparison using the Mann–Whitney U statistic, with one-sided testing (alternative: PANDAS > comparator). The Benjamini–Hochberg FDR procedure was applied separately within each comparison. To quantify the concordance of diagnostic performance across comparisons, the Spearman rank correlation coefficient was computed between AUC values obtained for PANDAS versus HC and PANDAS versus ASD across all proteins.

### Dual-threshold target classification

Protein targets were classified into three categories based on their diagnostic performance across comparisons: (1) dual discriminators (AUC ≥ 0.75 for both PANDAS versus HC and PANDAS vs. ASD), representing targets with potential PANDAS-specific diagnostic value; (2) HC-specific discriminators (AUC ≥ 0.80 for PANDAS vs. HC but < 0.65 for PANDAS vs. ASD), representing targets whose elevated reactivity in PANDAS is shared with ASD and thus likely reflects general neuroimmune dysregulation rather than PANDAS-specific autoimmunity; and (3) ASD-specific discriminators (AUC ≥ 0.80 for PANDAS vs. ASD but < 0.65 for PANDAS vs. HC), representing targets where ASD subjects show distinctly lower reactivity than both PANDAS and HC groups.

### Multi-protein panel classifiers for all comparisons

L2-regularized logistic regression classifiers with nested LOOCV (as described above) were trained for each of the three comparisons independently. Panel sizes of *k* = 5, 10, 20, and 50 features were evaluated. For the PANDAS versus all non-PANDAS comparison, the combined ASD + HC group served as the negative class, testing the clinically relevant scenario of identifying PANDAS within a mixed population of neuropsychiatric and healthy controls.

### Identification of PANDAS-specific autoantibody targets and functional enrichment analysis

To identify autoantibody targets unique to PANDAS, proteins from the secondary NAPPA screen (1,235 targets) were classified by group-specific reactivity. A protein was designated as PANDAS-specific if it showed a positive autoantibody response (MNI > 2) in at least one PANDAS subject and in none of the HC or ASD subjects. Analogous definitions were applied to identify HC-specific and ASD-specific targets. Targets positive in subjects from two or more diagnostic groups were classified as shared. Functional enrichment of PANDAS-specific targets was assessed using one-sided Fisher’s exact tests. Nine biologically relevant functional categories were defined based on Gene Ontology annotations and established gene function databases (UniProt, NCBI Gene, Human Protein Atlas): transcription factors/co-regulators, chromatin/epigenetic modification, immune regulation/cytokines, signal transduction (kinase/phosphatase), ubiquitin–proteasome system, cytoskeleton/motor proteins, apoptosis regulators, vesicle trafficking/endocytosis, and neural development/function. Proteins in the secondary screen background were mapped to 1,225 unique genes, and each was classified into applicable categories based on established gene function. For each category, a 2 × 2 contingency table was constructed comparing the proportion of PANDAS-specific targets in the category to the proportion expected by chance given the category’s representation in the full secondary screen background. *p*-values were corrected for multiple testing using the Benjamini–Hochberg FDR procedure across all nine categories tested. Additional comprehensive enrichment analysis with the 117 protein targets unique to PANDAS was conducted with the Database for Annotation, Visualization, and Integrated Discovery (DAVID) tool^2^ (Huang da et al., 2009; Sherman et al., 2022).

### Protein–protein interaction network and subcellular localization analysis

Protein–protein interaction networks were constructed using STRING v12^3^ (Szklarczyk et al., 2025) with a medium confidence threshold (score ≥ 0.4) and visualized in Cytoscape v3.x using the STRING app (Doncheva et al., 2023). Subcellular localization confidence scores were retrieved from the COMPARTMENTS database (Binder et al., 2014), which integrates curated annotations, experimental data from the Human Protein Atlas, text mining, and sequence-based predictions. To further evaluate the potential for direct pathogenic interaction between circulating autoantibodies and their target proteins, PANDAS-specific targets were classified by subcellular localization into four categories: cell surface/membrane receptor, secreted/extracellular, nuclear, and cytoplasmic. Localization assignments were based on UniProt subcellular localization annotations and Human Protein Atlas data. Targets were further dichotomized into “antibody-accessible” (cell surface + secreted, directly reachable by circulating IgG without requiring cell membrane disruption) and “intracellular” (nuclear + cytoplasmic, accessible only following cell damage or unconventional antigen presentation).

## Results

### Patient demographics

The ASD group had a significantly lower age compared to healthy controls (HC), but the age of PANDAS subjects did not differ between the HC or ASD groups. Representation of females was higher in our PANDAS cohort compared to HC or ASD groups, which had comparable distribution of sex (Table 1). One epidemiological study with a small cohort of Italian children suggested a higher incidence of PANDAS in males (Leonardi et al., 2023). Thus, we used the Mann–Whitney U test with FDR multiple testing correction in R (*tidyverse*) to test whether sex affected the normalized intensity of any protein target among PANDAS subjects in our initial screen of 15,006 full-length proteins. None of the targets reached a significant difference between male and female PANDAS subjects at P_adj_ < 0.1 (Figure 1B).

### Neurofilament light chain (NfL) assay

**Figure 2:**
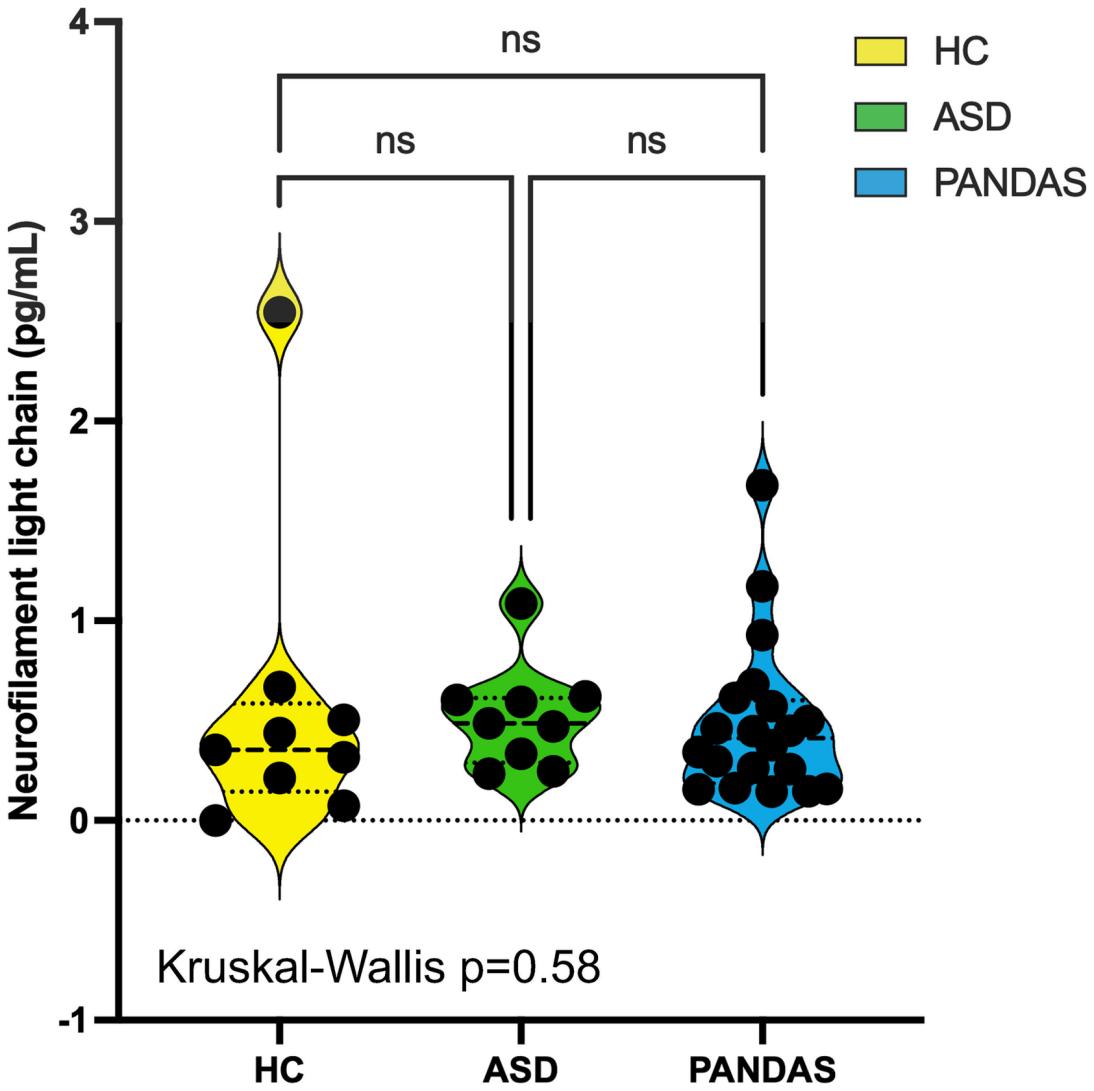
Serum neurofilament light chain is not elevated in ASD or PANDAS patients. Nine serum samples from the HC group (*n* = 9), ASD (*n* = 9), and PANDAS (*n* = 20) were analyzed for the NfL concentration using the Simoa™ NF-Light® kit (Quanterix). No statistical significance was observed before or after the elimination of statistically identified outliers.

NfL concentrations in the HC group did not pass the normality tests, and the *p* value of the Kruskal–Wallis test was 0.58, indicating no difference between groups. The ROUT method (*Q* = 1%) identified one outlier in the HC and one in the PANDAS groups. No description of any neuropsychiatric or neuroinflammatory events was reported for the HC outlier. The identified outlier in the PANDAS group (1.679 pg/mL; sample #PS31) was a subject with the latest age of onset in our cohort (14 years). The subject also had the most extensive history of neuropsychiatric disease among our PANDAS subjects and was the only one who, following the initial acute onset of extreme and aggressive OCD behavior, was repeatedly admitted to inpatient behavioral hospitals, where the subject was described as bipolar. The patient completed an extended Partial Hospitalization Program (PHP) and Intensive Outpatient Program (IOP) and received IVIG therapy around the time of sample collection. Age of onset, severity of symptoms, and the duration from the onset to sample collection (2.29 years) may be the reason for the higher serum NfL concentration. Elimination of the two outliers allowed each group to pass the Kolmogorov–Smirnov normality test, but one-way ANOVA still did not identify statistical differences between groups (*p* = 0.31). Figure 2 depicts all data points and the result of the Kruskal–Wallis test on the complete dataset (such as the two outliers).

Comparison to the Autoimmune Brain Panel

**Figure 3:**
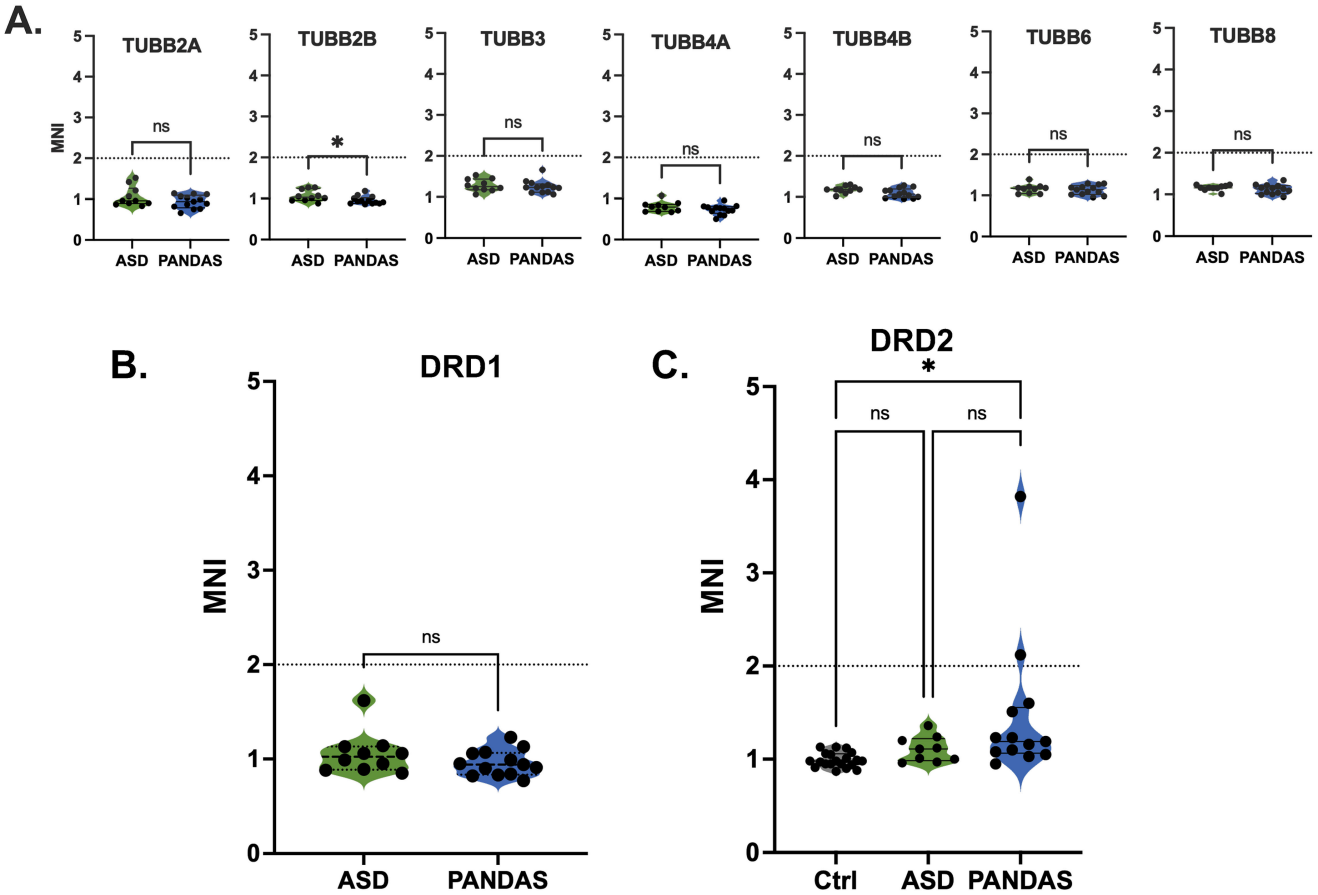
Autoreactivity against targets associated with the Autoimmune Brain Panel (Moleculera) in NAPPA array screening. Autoreactivity toward tubulin isoforms evaluated in the primary screen [PANDAS vs. ASD; panel **(A)**] or DRD1 dopamine receptor [panel **(B)**] did not identify them as a target of autoreactive IgG, and they did not advance to the secondary screening. Mean MNI was significantly lower in PANDAS subjects for the TUBB2B isoform. **(C)** Two out of 13 PANDAS subjects showed autoreactivity toward DRD2, which was sufficient to detect a statistically significant difference between MNI in healthy controls (Ctrl) and PANDAS, but not between ASD and PANDAS (1-way ANOVA followed by Tukey multiple comparison test; * *p* < 0.011).

The Autoimmune Brain Panel tests for autoantibodies toward dopamine D1 receptor (D1R), dopamine D2L receptor (D2LR), lysoganglioside-GM1, and tubulin. The fifth component of the test measures the effects of the patient’s serum on the activity of the CaMKII in human brain cells *in vitro* (Shimasaki et al., 2020). Of the four putative autoantibody targets, we did not screen for autoreactivity toward lysoganglioside-GM1, which is a glycolipid. In our PANDAS cohort, we did not observe increased autoreactivity toward any isoform of tubulin, with reactivity toward TUBB2B decreased among PANDAS subjects (Figure 3A). Anti-DRD1 signal was not statistically different between ASD and PANDAS in the initial screening (Figure 3B); therefore, we concluded that DRD1 autoantibodies do not discriminate between PANDAS and ASD, and DRD1 was not included in the subarrays used in the secondary screen. We confirmed generally higher DRD2 autoreactivity in PANDAS subjects compared to the HC, but in the secondary screen, it also did not discriminate between PANDAS and ASD (Figure 3C).

### Individual autoantibody targets show moderate but non-significant diagnostic performance

**Figure 4:**
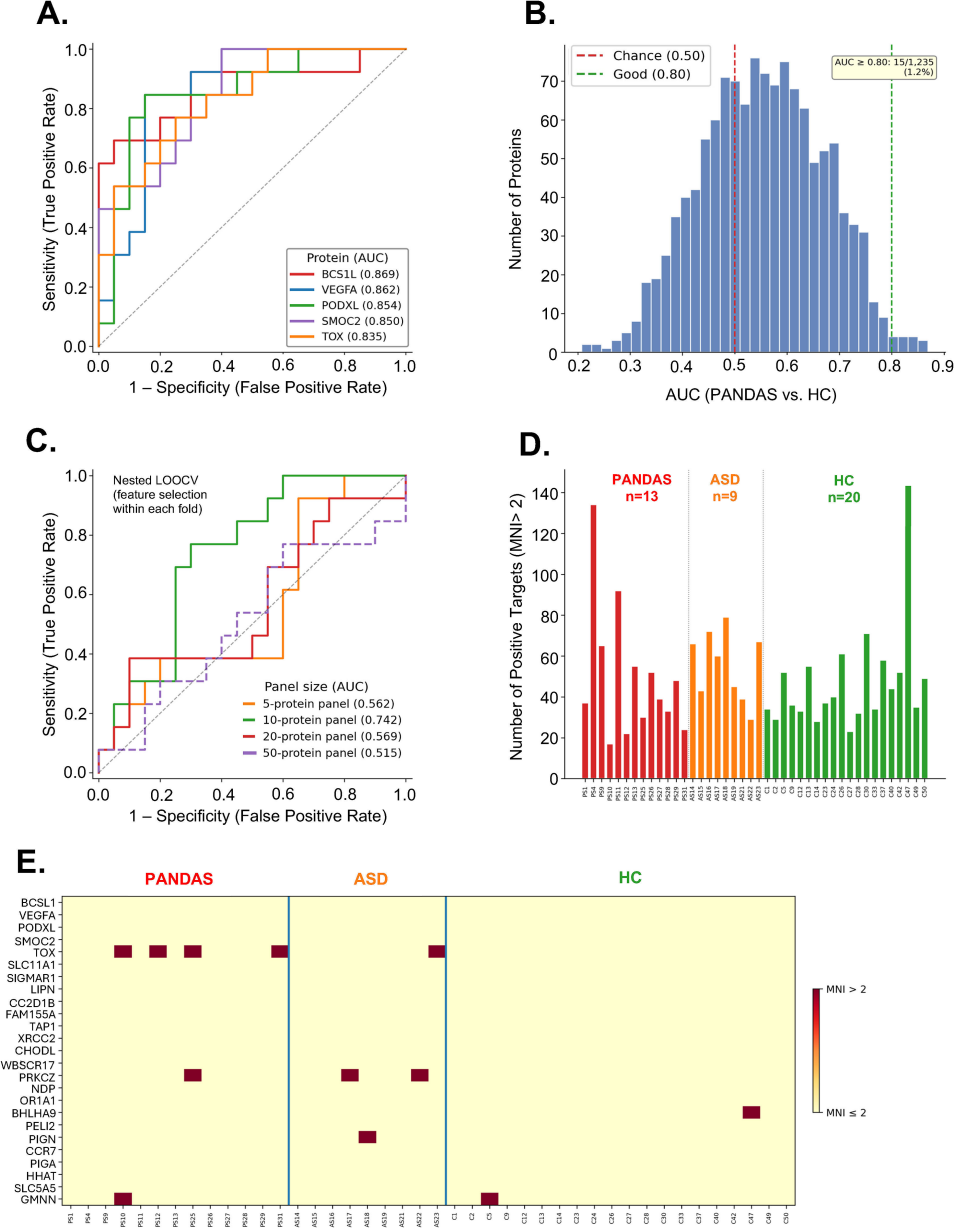
Diagnostic performance analysis of NAPPA autoantibody data. **(A)** ROC curves for the top five individual protein targets discriminating PANDAS from HC, with AUC values in parentheses. The dashed diagonal line represents chance performance (AUC = 0.50). **(B)** Distribution of AUC values across all 1,235 proteins; red and green dashed lines indicate chance and good performance thresholds, respectively. Only 16 proteins (1.3%) achieved AUC ≥ 0.80. **(C)** ROC curves for multi-protein panel classifiers (5, 10, 20, and 50 features) using nested LOOCV with feature selection performed exclusively within each cross-validation fold. **(D)** Number of positive autoantibody targets (MNI > 2) per subject across PANDAS, ASD, and HC groups, demonstrating comparable autoantibody burden across groups. **(E)** Binary heatmap of the top 25 PANDAS-enriched proteins across all subjects, illustrating the sparse, patient-specific nature of autoantibody positivity.

ROC analysis was performed for all 1,235 protein targets to assess their individual diagnostic utility for distinguishing PANDAS (*n* = 13) from HC (*n* = 20) subjects (Supplementary File 1). The best-performing individual targets included BCS1L (AUC = 0.869), VEGFA (AUC = 0.862), PODXL (AUC = 0.854), SMOC2 (AUC = 0.850), and TOX (AUC = 0.835) (Figure 4A; Table 2). However, no individual protein achieved statistical significance after correction for multiple testing (lowest FDR *q* = 0.126 for BCS1L), and only 21 of 1,235 targets had FDR *q* < 0.20. The distribution of AUC values across all proteins showed that the majority clustered near the chance level of 0.50, with only 16 proteins (1.3%) achieving an AUC ≥ 0.80 (Figure 4B). Notably, most of the top-ranked proteins showed elevated mean MNI in PANDAS relative to HC, but without exceeding the MNI > 2 positivity threshold in either group, indicating that the discriminatory signal was driven by subtle distributional shifts in MNI rather than clearly positive autoantibody responses. The exception was TOX, which was positive in 4 of 13 PANDAS subjects (30.8%) and in none of the 20 HC subjects.

**Table 2:** Top 20 autoantibody targets ranked by AUC for PANDAS vs. HC discrimination.

Protein	AUC	*p*-value	FDR *q*	PANDAS +	HC +	PANDAS MNI	HC MNI
BCS1L	0.869	2.17 × 10^−4^	0.126	0/13 (0%)	0/20 (0%)	0.96	0.87
VEGFA	0.862	2.86 × 10^−4^	0.126	0/13 (0%)	0/20 (0%)	1.07	0.95
PODXL	0.854	3.74 × 10^−4^	0.126	0/13 (0%)	0/20 (0%)	1.04	0.87
SMOC2	0.850	4.27 × 10^−4^	0.126	0/13 (0%)	0/20 (0%)	1.10	0.90
TOX	0.835	7.19 × 10^−4^	0.126	4/13 (30.8%)	0/20 (0%)	3.41	0.97
SLC11A1	0.835	7.19 × 10^−4^	0.126	0/13 (0%)	0/20 (0%)	1.01	0.84
SIGMAR1	0.835	7.19 × 10^−4^	0.126	0/13 (0%)	0/20 (0%)	1.01	0.91
LIPN	0.831	8.16 × 10^−4^	0.126	0/13 (0%)	0/20 (0%)	0.96	0.86
CC2D1B	0.827	9.25 × 10^−4^	0.127	0/13 (0%)	0/20 (0%)	1.14	0.90
TAP1	0.819	1.18 × 10^−3^	0.133	0/13 (0%)	0/20 (0%)	1.02	0.92
FAM155A	0.819	1.18 × 10^−3^	0.133	0/13 (0%)	0/20 (0%)	1.06	0.94
CHODL	0.812	1.51 × 10^−3^	0.143	0/13 (0%)	0/20 (0%)	0.97	0.91
XRCC2	0.812	1.51 × 10^−3^	0.143	0/13 (0%)	0/20 (0%)	1.08	0.96
WBSCR17	0.808	1.70 × 10^−3^	0.150	0/13 (0%)	0/20 (0%)	1.22	1.01
PRKCZ	0.804	1.91 × 10^−3^	0.157	1/13 (7.7%)	0/20 (0%)	1.32	1.02
NDP	0.792	2.70 × 10^−3^	0.199	0/13 (0%)	0/20 (0%)	0.97	0.88
OR1A1	0.788	3.03 × 10^−3^	0.199	0/13 (0%)	0/20 (0%)	0.90	0.84
BHLHA9	0.787	3.20 × 10^−3^	0.199	0/13 (0%)	1/20 (5%)	1.09	1.04
PELI2	0.785	3.38 × 10^−3^	0.199	0/13 (0%)	0/20 (0%)	1.01	0.92
CCR7	0.785	3.38 × 10^−3^	0.199	0/13 (0%)	0/20 (0%)	0.99	0.90

AUC, area under the ROC curve; FDR *q*, Benjamini-Hochberg false discovery rate adjusted *q*-value; PANDAS +, number (%) of PANDAS subjects with MNI > 2; HC +, number (%) of HC subjects with MNI > 2; P MNI, mean MNI in PANDAS; HC MNI, mean MNI in HC.

### Multi-protein panel classifiers did not achieve reliable discrimination under proper cross-validation

To determine whether combining multiple targets into a diagnostic panel could overcome the limitations of individual proteins, logistic regression classifiers with panel sizes of *k* = 5, 10, 20, and 50 features were evaluated using nested LOOCV (Figure 4C). The 10-protein panel yielded the highest cross-validated AUC of 0.742 (sensitivity = 0.769, specificity = 0.700), while the 5-protein panel achieved an AUC of 0.562, the 20-protein panel an AUC of 0.569, and the 50-protein panel an AUC of 0.515. These results contrast sharply with naïve analyses in which feature selection is performed on the entire dataset before evaluation, which produced inflated AUC values of up to 0.946, demonstrating the critical importance of proper cross-validation in high-dimensional settings where the number of features (*p* = 1,235) greatly exceeds the number of samples (*n* = 33). Permutation testing (500 permutations) of the 20-protein panel yielded a non-significant *p*-value of 0.14 (null AUC distribution: mean = 0.417, SD = 0.147), further indicating that the observed panel performance does not exceed chance expectation.

### High heterogeneity of autoantibody profiles underlies poor diagnostic performance

The limited diagnostic performance of both individual targets and multi-protein panels is explained by the profound heterogeneity of autoantibody profiles among PANDAS subjects. Of 399 proteins showing positivity (MNI ≥ 2) in at least one PANDAS subject, 269 (67.4%) were positive in only a single PANDAS patient, 78 (19.5%) in exactly two patients, and only 53 (13.3%) in three or more patients. A single protein was positive in 12 of 13 PANDAS patients, but no protein was universally positive across all 13. The number of positive targets per PANDAS patient ranged from 17 to 134 (mean = 49.8, median = 39.0), further demonstrating the individualized nature of autoantibody responses (Figure 4D,E; Supplementary File 2). The mean number of hits per subject did not differ meaningfully between groups (PANDAS: 49.8; ASD: 55.6; HC: 47.4) and the total hit count per subject performed at chance level as a diagnostic measure (AUC = 0.483), confirming that the autoantibody burden per se does not distinguish PANDAS from controls. Analysis of target overlap across groups revealed that 117 of 399 PANDAS-positive targets (29.5%) were not detected in either ASD or HC subjects, while 134 targets (33.8%) were shared across all three groups, indicating substantial background autoantibody reactivity common to all individuals.

### Autoantibody targets that discriminate PANDAS from HC largely fail to discriminate PANDAS from ASD

**Figure 5:**
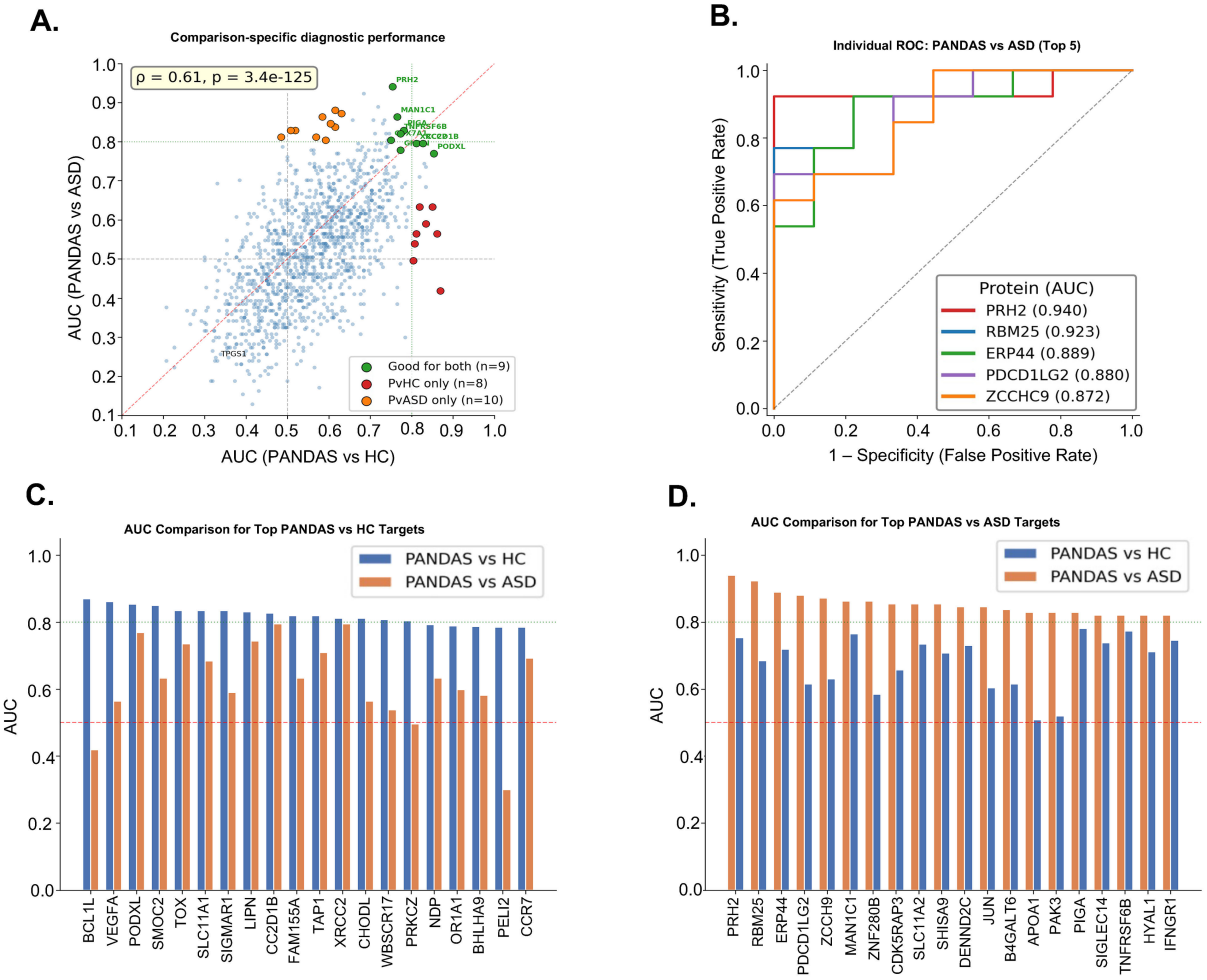
Three-group diagnostic performance analysis. **(A)** Scatter plot of individual protein AUC values for PANDAS versus HC (*x*-axis) versus PANDAS versus ASD (*y*-axis). Green points indicate dual discriminators (AUC ≥ 0.75 in both comparisons, *n* = 9); red points indicate HC-specific discriminators (AUC ≥ 0.80 vs. HC, <0.65 vs. ASD, *n* = 8); and orange points indicate ASD-specific discriminators (AUC ≥ 0.80 vs. ASD, <0.65 vs. HC, *n* = 10). Spearman correlation shown. **(B)** ROC curves for the top five individual targets discriminating PANDAS from ASD. **(C)** Paired AUC comparison for the top 20 PANDAS versus HC targets, showing their performance when tested against ASD (orange bars). Many targets exhibit substantial AUC drops, indicating shared reactivity between PANDAS and ASD. **(D)** Paired AUC comparison for the top 20 PANDAS versus ASD targets, showing their performance against HC.

To determine whether PANDAS-associated autoantibody targets provide disease-specific diagnostic information or instead reflect broader neuroimmune dysregulation, ROC analysis was performed for PANDAS versus ASD in addition to the primary PANDAS versus HC comparison. Individual target AUC values showed moderate concordance between the two comparisons (Spearman *ρ* = 0.61, *p* = 3.4 × 10^−125^; Figure 5A), indicating that a protein’s ability to discriminate PANDAS from HC is a poor predictor of its ability to discriminate PANDAS from ASD. This discordance was most pronounced among the highest-ranked PANDAS versus HC targets. Of the top 20 PANDAS versus HC discriminators, 10 showed AUC drops exceeding 0.10 when tested against ASD, and 8 of 16 proteins with AUC ≥ 0.80 for PANDAS versus HC fell below AUC 0.65 for PANDAS versus ASD (Table 3). The most dramatic example was BCS1L, the best individual PANDAS vs. HC discriminator (AUC = 0.869), which dropped to AUC = 0.419 against ASD—indicating that ASD subjects had higher BCS1L reactivity than PANDAS subjects. Similarly, VEGFA (0.862 → 0.564), SMOC2 (0.850 → 0.632), SIGMAR1 (0.835 → 0.590), and PRKCZ (0.804 → 0.496) all showed substantial performance collapse (Figure 5C). These targets thus appear to detect autoantibody reactivity that is shared between PANDAS and ASD, distinguishing either condition from healthy individuals but unable to differentiate between the two neuropsychiatric groups.

**Table 3:** Comparison-specific autoantibody targets demonstrating discordant diagnostic performance.

Protein	AUC (PvHC)	*p*-value (PvHC)	AUC (PvASD)	*p*-value (PvASD)	Discriminates	Interpretation
BCS1L	0.869	2.17 × 10^−4^	0.419	8.06 × 10^−1^	HC only	Shared with ASD
VEGFA	0.862	2.86 × 10^−4^	0.564	1.93 × 10^−1^	HC only	Shared with ASD
SMOC2	0.850	4.27 × 10^−4^	0.632	8.82 × 10^−2^	HC only	Shared with ASD
SIGMAR1	0.835	7.19 × 10^−4^	0.590	1.27 × 10^−1^	HC only	Shared with ASD
PRKCZ	0.804	1.91 × 10^−3^	0.496	4.68 × 10^−1^	HC only	Shared with ASD
WBSCR17	0.808	1.70 × 10^−3^	0.538	2.52 × 10^−1^	HC only	Shared with ASD
PRH2	0.754	7.28 × 10^−3^	0.940	3.30 × 10^−4^	Both	PANDAS-specific
PDCD1LG2	0.615	5.35 × 10^−2^	0.880	1.65 × 10^−3^	ASD only	ASD has lower reactivity
ZNF280B	0.585	8.69 × 10^−2^	0.863	2.52 × 10^−3^	ASD only	ASD has lower reactivity
JUN	0.604	6.73 × 10^−2^	0.846	3.78 × 10^−3^	ASD only	ASD has lower reactivity
PAK3	0.519	3.25 × 10^−1^	0.829	5.57 × 10^−3^	ASD only	ASD has lower reactivity
APOA1	0.508	3.55 × 10^−1^	0.829	5.58 × 10^−3^	ASD only	ASD has lower reactivity

HC-specific discriminators (AUC ≥ 0.80 vs. HC, < 0.65 vs. ASD) likely reflect autoantibody reactivity shared between PANDAS and ASD. ASD-specific discriminators (AUC ≥ 0.80 vs. ASD, < 0.65 vs. HC) identify targets where ASD subjects show distinctly lower reactivity than both PANDAS and HC (PvASD – PANDAS vs. ASD, PvHC – PANDAS vs. HC).

### PANDAS versus ASD discrimination requires a distinct set of targets, and very few targets show dual discriminatory capacity

The top targets for PANDAS versus ASD discrimination were largely non-overlapping with the PANDAS versus HC target set (Figures 5B,D). The strongest PANDAS versus ASD discriminator was PRH2 (AUC = 0.940), which showed fair but reduced performance against HC (AUC = 0.754). Other top PANDAS versus ASD targets, such as RBM25 (AUC = 0.923 vs. ASD, 0.685 vs. HC), PDCD1LG2 (0.880, 0.615), and ZNF280B (0.863, 0.585), performed substantially worse against HC (Figure 5D). Ten proteins with AUC ≥ 0.80 for PANDAS versus ASD fell below 0.65 for PANDAS versus HC. No individual protein achieved FDR *q* < 0.05 for the PANDAS versus ASD comparison (minimum FDR *q* = 0.330), consistent with the heterogeneity observed in the PANDAS versus HC analysis. Only 25 proteins achieved AUC ≥ 0.80 for PANDAS versus ASD, compared to 16 for PANDAS versus HC, with a total of 106 reaching nominal *p* < 0.05 (vs. 204 for PANDAS vs. HC). Only nine proteins achieved AUC ≥ 0.75 in both comparisons simultaneously (Table 4): CC2D1B, XRCC2, PIGA, GMNN, TNFRSF6B, PODXL, MAN1C1, PRH2, and COX7A1. These dual discriminators represent the most promising PANDAS-specific candidates, as they detect autoantibody reactivity that is elevated in PANDAS relative to both healthy individuals and ASD subjects. However, none achieved FDR significance in either comparison, and the modest AUC values (minimum AUC 0.75–0.80) indicated limited standalone diagnostic utility. Among these, the immunologically most notable targets include TNFRSF6B (decoy receptor for TRAIL/FasL involved in immune regulation), PIGA (GPI anchor biosynthesis, deficiency of which causes paroxysmal nocturnal hemoglobinuria), and TAP1-associated antigen processing pathways.

**Table 4:** Dual-discriminator autoantibody targets with AUC ≥ 0.75 for both PANDAS versus HC (PvHC) and PANDAS versus ASD (PvASD) comparisons.

Protein	AUC (PvHC)	*p*-value (PvHC)	FDR *q* (PvHC)	AUC (PvASD)	*p*-value (PvASD)	FDR *q* (PvASD)	Min AUC
CC2D1B	0.827	9.25 × 10^−4^	0.127	0.795	1.52 × 10^−2^	0.476	0.795
XRCC2	0.812	1.51 × 10^−3^	0.143	0.795	1.52 × 10^−2^	0.476	0.795
PIGA	0.781	3.78 × 10^−3^	0.212	0.829	5.58 × 10^−3^	0.416	0.781
GMNN	0.773	4.70 × 10^−3^	0.213	0.778	1.17 × 10^−2^	0.453	0.773
TNFRSF6B	0.773	4.70 × 10^−3^	0.213	0.821	6.74 × 10^−3^	0.416	0.773
PODXL	0.854	3.74 × 10^−4^	0.126	0.769	1.34 × 10^−2^	0.460	0.769
MAN1C1	0.765	5.84 × 10^−3^	0.228	0.863	2.52 × 10^−3^	0.382	0.765
PRH2	0.754	7.28 × 10^−3^	0.249	0.940	3.30 × 10^−4^	0.330	0.754
COX7A1	0.750	8.04 × 10^−3^	0.260	0.803	8.04 × 10^−3^	0.416	0.750

HC-specific discriminators (AUC ≥ 0.80 vs. HC, < 0.65 vs. ASD) likely reflect autoantibody reactivity shared between PANDAS and ASD. ASD-specific discriminators (AUC ≥ 0.80 vs. ASD, < 0.65 vs. HC) identify targets where ASD subjects show distinctly lower reactivity than both PANDAS and HC (PvASD, PANDAS vs. ASD; PvHC, PANDAS vs. HC).

### Panel-based classifiers show comparison-dependent performance

Multi-protein panel classifiers with nested LOOCV showed marked variation in performance depending on the comparison group (Table 5). Against HC, the best panel achieved AUC = 0.742 (*k* = 10, sensitivity = 0.769, specificity = 0.700). Against ASD, all panel sizes performed at or below chance level (AUC = 0.222–0.342), reflecting the smaller ASD sample size (*n* = 9) and the lack of consistent discriminatory features between these two neuropsychiatric groups. For the clinically most relevant comparison—PANDAS versus all non-PANDAS subjects (ASD + HC combined, *n* = 29)—the 20-feature panel yielded the highest AUC of 0.777 (sensitivity = 0.923, specificity = 0.724), benefiting from the larger comparator group. However, this performance remains below the threshold typically required for clinical diagnostic utility (AUC ≥ 0.90). The failure of panel classifiers to distinguish PANDAS from ASD is particularly significant because it demonstrates that the autoantibody heterogeneity problem is compounded in the differential diagnosis setting. Not only do individual PANDAS patients have different autoantibody targets (as shown above), but the comparison group itself influences which targets appear informative, meaning that a panel optimized for one clinical question may be uninformative for another.

**Table 5:** Cross-validated panel classifier performance (nested LOOCV) across three diagnostic comparisons.

Comparison	*k* = 5	*k* = 10	*k* = 20	*k* = 50	*k* = 5	*k* = 10	*k* = 20	*k* = 50
PANDAS vs. HC (*n* = 13 vs. 20)	0.562	0.742	0.569	0.515	0.92/0.35	0.77/0.70	0.39/0.90	0.77/0.40
PANDAS vs. ASD (*n* = 13 vs. 9)	0.342	0.222	0.333	0.325	1.0/0.11	0.00/1.0	0.08/1.0	0.92/0.11
PANDAS vs All (*n* = 13 vs. 29)	0.127	0.690	0.777	0.541	0.08/0.93	0.69/0.76	0.92/0.72	0.54/0.79

AUC, area under the ROC curve; *k*, number of features selected by *χ*^2^ test within each cross-validation fold; Sens/Spec, sensitivity and specificity at the Youden’s J optimal threshold.

To assess whether the failure of panel classifiers against ASD reflected insufficient statistical power (*n* = 9) or genuine biological overlap, we subsampled nine HCs from the full cohort 500 times and repeated the nested LOOCV analysis. The resulting AUC distribution (median = 0.556, SD = 0.178) showed substantial instability with only nine comparators, indicating that reduced sample size contributes to poor classifier performance. However, examination of individual target reactivity provided direct evidence for shared autoantibody profiles: 18 of the 20 strongest PANDAS versus HC discriminators showed higher mean MNI in ASD than in HC subjects, indicating that ASD subjects exhibit PANDAS-like autoantibody reactivity rather than HC-like profiles (Supplementary Figure S1). These findings suggest that the poor discriminatory performance against ASD reflects both limited statistical power and genuine biological overlap in autoantibody reactivity between PANDAS and ASD.

### PANDAS-specific targets are enriched for neuronal, epigenetic, and immune-regulatory functions

**Figure 6:**
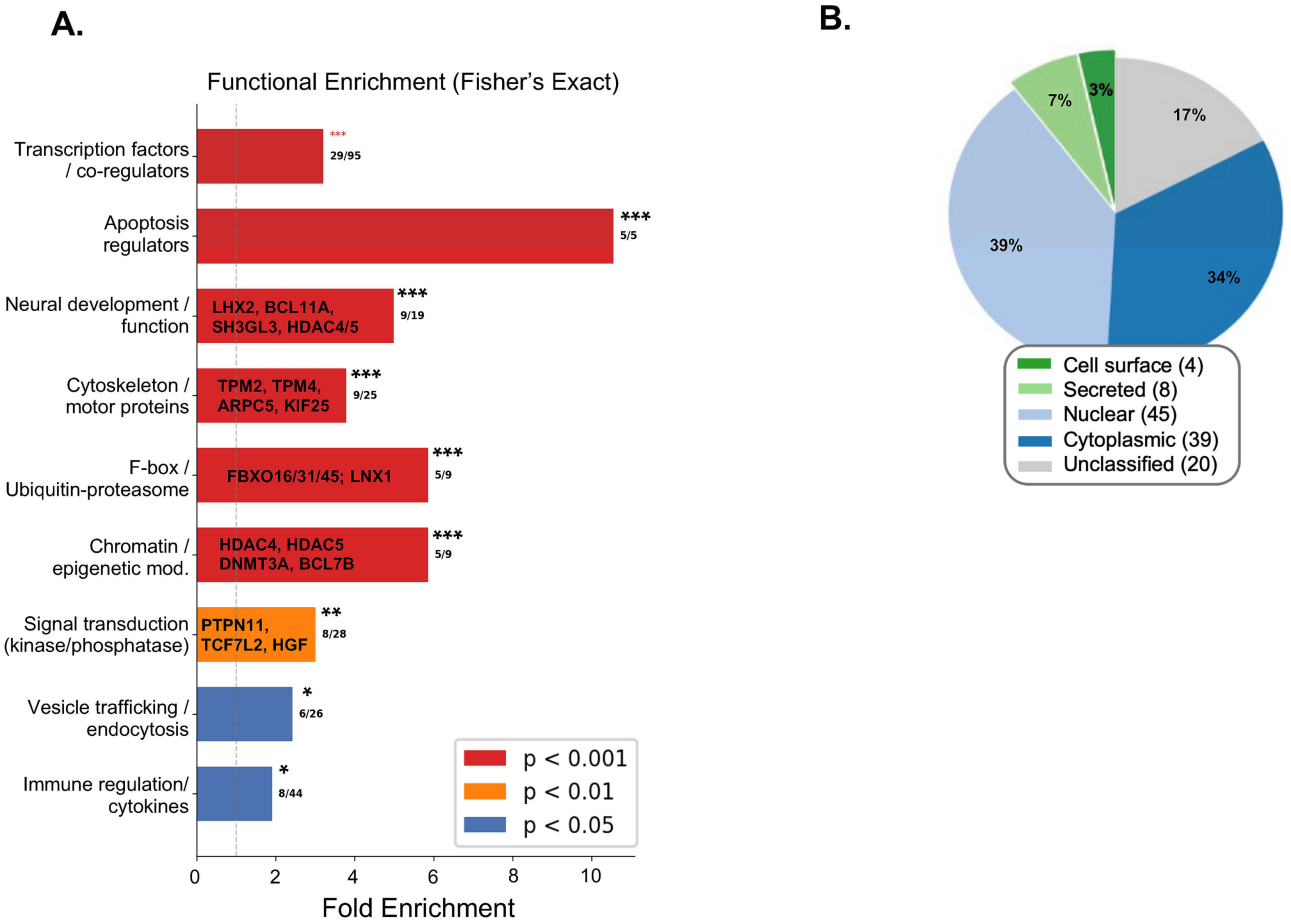
Functional characterization of PANDAS-specific autoantibody targets. **(A)** Fold-enrichment of nine biological categories among 117 PANDAS-specific targets relative to the 1,225-gene secondary screen background, assessed by Fisher’s exact test. Numbers indicate observed/background gene counts per category. All categories reached FDR significance (asterisks: *p < 0.05, **p < 0.01, ***p < 0.001). Selected proteins with potential relevance for PANDAS pathogenesis are listed within bars. **(B)** Subcellular localization and distribution of PANDAS-specific targets. Only 10.3% of targets are directly accessible to circulating autoantibodies (cell surface + secreted), while 72.4% are intracellular.

Functional enrichment analysis revealed that PANDAS-specific autoantibody targets were significantly enriched across several biological categories (Table 6; Figure 6A). The most statistically significant enrichment was observed for transcription factors and co-regulators (29 of 95 background genes, 3.2-fold enrichment, FDR *q* = 8.7 × 10^−9^), indicating a marked overrepresentation of gene-regulatory proteins among PANDAS-specific targets. This included LIM-homeodomain transcription factors critical for neural development (LHX2, LHX8), forkhead-box family members (FOXL1), Wnt pathway effectors (TCF7L2), and multiple zinc finger proteins (ZNF687, ZNF395, ZNF280B, ZNF518A, and ZNF572). The strongest fold-enrichment was observed for apoptosis regulators (5 of 5 background genes, 10.6-fold, FDR *q* = 3.2 × 10^−5^), with all apoptosis-related proteins on the array—such as the anti-apoptotic regulator BCL2, the neurodevelopmental transcription factor BCL11A, the SWI/SNF chromatin remodeling component BCL7B, and the tumor suppressor DLEU7—being exclusively targeted in PANDAS. Autoantibodies against BCL2 are of particular interest, as disruption of BCL2-mediated survival signaling could promote neuronal apoptosis in affected brain regions.

**Table 6:** Functional enrichment analysis of PANDAS-specific autoantibody targets.

Functional category	*k*	*K*	Expected	Fold	*p*-value	FDR *q*
Transcription factors/co-regulators	29	95	9.0	3.22	9.68 × 10^−10^	8.71 × 10^−9^
Apoptosis regulators	5	5	0.5	10.56	7.03 × 10^−6^	3.17 × 10^−5^
Neural development/function	9	19	1.8	5.00	1.86 × 10^−5^	5.57 × 10^−5^
Cytoskeleton/motor proteins	9	25	2.4	3.80	2.51 × 10^−4^	5.64 × 10^−4^
F-box/Ubiquitin-proteasome	5	9	0.9	5.87	6.47 × 10^−4^	9.71 × 10^−4^
Chromatin/epigenetic modification	5	9	0.9	5.87	6.47 × 10^−4^	9.71 × 10^−4^
Signal transduction (kinase/phosphatase)	8	28	2.7	3.02	3.13 × 10^−3^	4.02 × 10^−3^
Vesicle trafficking/endocytosis	6	26	2.5	2.44	3.00 × 10^−2^	3.38 × 10^−2^
Immune regulation/cytokines	8	44	4.2	1.92	4.91 × 10^−2^	4.91 × 10^−2^

*k*, number of PANDAS-specific targets in category; *K*, number of genes in category in the secondary screen background (1,225 unique genes); Expected, number of PANDAS-specific targets expected by chance; Fold, fold-enrichment (k/Expected); *p*-value, one-sided Fisher’s exact test; FDR *q*, Benjamini-Hochberg corrected q-value.

Neural development and function proteins were 5.0-fold enriched (9 of 19, FDR *q* = 5.6 × 10^−5^). This category included BCL11A, whose haploinsufficiency causes Dias–Logan syndrome, characterized by intellectual disability and developmental delay; LHX2 and LHX8, LIM-homeodomain factors essential for cortical and basal forebrain development, respectively; SH3GL3 (endophilin A3), which mediates synaptic vesicle recycling at nerve terminals; and TULP3, a ciliary signaling protein with roles in neural tube patterning. The enrichment of neuronal proteins among PANDAS-specific autoantibody targets provides a direct molecular link between the observed autoimmune response and potential CNS dysfunction.

Chromatin and epigenetic regulators were 5.9-fold enriched (5 of 9, FDR *q* = 9.7 × 10^−4^), such as HDAC4 and HDAC5—class IIa histone deacetylases that play critical roles in synaptic plasticity, memory formation, and neuronal gene expression and are highly expressed in the basal ganglia—and DNMT3A, a *de novo* DNA methyltransferase whose germline mutations cause Tatton-Brown–Rahman syndrome with intellectual disability. The concurrent enrichment of epigenetic regulators and neural developmental factors suggests that PANDAS-associated autoimmunity may converge on transcriptional programs governing neuronal function.

Additional significantly enriched categories included cytoskeleton/motor proteins (9 of 25, 3.8-fold, FDR *q* = 5.6 × 10^−4^), encompassing tropomyosins (TPM2, TPM4), the ARP2/3 complex subunit ARPC5, and kinesin family members (KIF25, KLC4); the ubiquitin–proteasome system (5 of 9, 5.9-fold, FDR *q* = 9.7 × 10^−4^), with three F-box proteins (FBXO16, FBXO31, FBXO45) and the E3 ligase LNX1; signal transduction kinases and phosphatases (8 of 28, 3.0-fold, FDR *q* = 4.0 × 10^−3^), notably such as PTPN11 (SHP2), a key RAS-MAPK phosphatase whose germline mutations cause Noonan syndrome; vesicle trafficking (6 of 26, 2.4-fold, FDR *q* = 3.4 × 10^−2^); and immune regulation/cytokines (8 of 44, 1.9-fold, FDR *q* = 4.9 × 10^−2^), such as the MHC class II master regulator CIITA, the T cell checkpoint receptor LAG3, the neutrophil chemokine CXCL6, and prolactin (PRL). The results of comprehensive enrichment analysis using the DAVID tool can be found in Supplementary File 3.

### Majority of PANDAS-specific targets are intracellular proteins enriched for disordered regions

Subcellular localization analysis revealed that most PANDAS-specific autoantibody targets were intracellular proteins (Table 7; Figure 6B). Nuclear proteins constituted the largest fraction (38.8%), followed by cytoplasmic proteins (33.6%), while only 12 targets (10.3%) were directly accessible to circulating autoantibodies: four cell-surface receptors (OLR1, LAG3, ANO1, and NEU3) and eight secreted/extracellular proteins (CXCL6, HGF, PRL, OLFML2B, TLL2, SRGN, TCN2, and TGM1). 92/117 (77.8%) of protein targets specific to PANDAS were enriched for disordered regions (P_adj_ = 9.74E-05). To explore functional relationships among the PANDAS-specific autoantibody targets, we constructed a protein–protein interaction (PPI) network using the STRING database (v12) within Cytoscape (Supplementary Figure S2). The network revealed a prominent interconnected cluster centered on BCL2, which served as a major hub connecting several functionally related proteins, such as the histone deacetylases HDAC4 and HDAC5 (among the most frequently seropositive targets), the tyrosine phosphatase PTPN11/SHP2, hepatocyte growth factor (HGF), DNA methyltransferase DNMT3A, and the immune checkpoint receptor LAG3. Additional connected nodes included transcriptional regulators (RUNX2, TCF7L2, and CIITA), the hypoxia-responsive factor EPAS1/HIF-2α, and prolactin (PRL). A second, smaller cluster included the SWI/SNF chromatin remodeling components BCL11A and BCL7B linked to tropomyosins (TPM2, TPM4). Notably, the majority of identified targets remained as singletons or small disconnected components, suggesting that most of the autoantibodies, in their diversity, target a functionally diverse set of proteins rather than a single pathway.

**Table 7:** Subcellular localization of PANDAS-specific autoantibody targets.

Subcellular localization	Count	% of total
Cell surface/Membrane receptor	4	3.4%
Secreted/Extracellular	8	6.9%
Nuclear	45	38.8%
Cytoplasmic	39	33.6%
Unclassified	20	17.2%
Directly antibody-accessible (surface + secreted)	12	10.3%
Intracellular (nuclear + cytoplasmic)	84	72.4%

Assignments were based on UniProt and Human Protein Atlas annotations.

### Sensitivity analysis: impact of excluding sample PS31 (IVIG-treated)

Following identification that one PANDAS patient (PS31) received intravenous immunoglobulin (IVIG) therapy around the time of serum collection, we performed a sensitivity analysis to determine whether exogenous polyclonal antibodies from IVIG may have confounded the PANDAS-specific autoantibody profile. IVIG is pooled IgG from thousands of donors and contains broad reactivities to self-antigens; its presence in a patient sample could plausibly introduce false-positive signals on the NAPPA array. We therefore re-ran the entire downstream pipeline (PANDAS-specific target identification, patient distribution, and functional category enrichment), with PS31 excluded, using the remaining 12 PANDAS samples against the same 9 ASD and 20 HC controls. PANDAS-specific targets were redefined using the identical MNI > 2.0 positivity threshold applied in the original analysis, with the requirement that a protein be positive in ≥1 PANDAS patient and in 0 ASD and 0 HC samples. The sample set comprised 12 PANDAS (all original PS samples except PS31), 9 ASD, and 20 HCs. Functional category enrichment used Fisher’s exact test against the same 1,235-gene secondary-screen background, with categories and gene-category memberships identical to the primary analysis. BH-adjusted FDR was computed across the same set of tested categories.

#### Key finding: PS31 contributed minimally to the PANDAS-specific repertoire

Of the 117 PANDAS-specific targets identified in the original analysis, PS31 was positive for only four targets (3.4%): PLEKHM3, PPP5D1, ANO1, and CENPT (Supplementary Table S2). Of these, only three targets were lost upon PS31 exclusion—PPP5D1, ANO1, and CENPT—because PS31 was their sole positive patient (singletons). The fourth target, PLEKHM3, was also positive in PS9 and PS28 and therefore remained in the PANDAS-specific set after PS31 removal. Consequently, the sensitivity analysis yielded 114 PANDAS-specific targets (vs. 117 original), a reduction of only 2.6%.

#### Patient-level heterogeneity was unchanged

The profound patient-specific heterogeneity that characterized the original analysis was fully preserved: 103 of 114 targets (90.4%) were positive in only a single patient after PS31 exclusion, compared with 106 of 117 (90.6%) in the original analysis. Targets shared by two or more patients comprised 9.4% original and 9.6% after PS31 exclusion. The one target that shifted from three-patient to two-patient positivity was PLEKHM3 (originally PS9/PS28/PS31, now PS9/PS28). All other multi-patient targets—such as HDAC5, FATE1, BCL11A, BCL7B, GAB4, MED26, CXCL6, KLC4, PRKAB2, and DKKL1—were unaffected.

#### Functional category enrichment remained robust

All nine functional categories that reached statistical significance in the original enrichment analysis remained significant after PS31 exclusion, with fold-enrichment values and *p*-values that were essentially unchanged or marginally strengthened (Supplementary Table S3). The strongest categories—transcription factors (20/20 PANDAS targets, fold 3.30, *p* = 3.89 × 10^−22^), apoptosis regulators (4/4, fold 10.84, *p* = 6.90 × 10^−5^), neural development (9/19, fold 5.13, *p* = 1.31 × 10^−5^), and chromatin/epigenetic regulators (5/9, fold 6.02, *p* = 5.60 × 10^−4^)—all retained their directionality, magnitude, and statistical significance. None of the curated apoptosis, transcription factor, chromatin, immune regulation, neural development, or signal transduction targets depend on PS31 for their positive call. The core pathogenic interpretation—convergence of autoantibody targeting on nuclear transcription factors, apoptosis machinery, epigenetic regulators, and neural development proteins—was fully preserved.

## Discussion

PANDAS symptoms have been postulated to represent a psychiatric outcome of neuroinflammation resulting from group A streptococcal (GAS) in a genetically susceptible host. Preclinical murine studies (Platt et al., 2020; Wayne et al., 2023; Dileepan et al., 2016) and work with human and murine brain specimens (Xu et al., 2021; Xu et al., 2024; Frick et al., 2018) suggest that GAS infection may lead to IL17-mediated disruption of the blood–brain barrier, infiltration of the brain by GAS-specific pathogenic T cells, and the production of anti-GAS antibodies, which cross-react with cholinergic interneurons in the striatum through molecular mimicry.

The knowledge that immune dysregulation plays a role in PANDAS pathogenesis has been used to both diagnose (via the Autoimmune Brain Panel) and treat (non-steroidal anti-inflammatory drugs, corticosteroids, IVIg, and plasmapheresis) subjects with this condition. Despite the increasing awareness that dysfunction of the immune system (in the setting of preceding infection) is responsible for disease pathogenesis, clinicians are still lacking reliable and specific serological tools.

In our study, we took an unbiased approach to screening for autoantibodies in subjects diagnosed with PANDAS and contrasted the results with healthy controls and ASD with a two-fold goal. The first goal was to identify marker(s) of PANDAS, which individually or as a set, could provide a novel diagnostic approach. In this approach, we were agnostic to the disease pathogenesis, expression pattern, or cellular location of the protein target, with the assumption that “bystander” autoantibody targets may still be of diagnostic value. The second goal was to search for targets of PANDAS-associated IgG that additionally may shed more light on the disease pathogenesis. The focus of well-diagnosed PANDAS cases, instead of a broader PANS category, was motivated by the anticipated relative homogeneity of autoimmune response as a consequence of GAS infection.

Except for the DRD2 dopamine receptor, we could not identify commonalities in our screening with targets included in the Moleculera’s Autoimmune Brain Panel. This finding needs to be interpreted with caution as methodical differences in sample collection and assay principles do not allow us to question or dismiss the diagnostic value of the Moleculera’s laboratory-developed test.

Our systematic evaluation of diagnostic performance revealed that neither individual autoantibody targets nor multi-protein panel classifiers achieved clinically reliable discrimination of PANDAS. While 16 individual proteins reached an AUC ≥ 0.80 for PANDAS compared to healthy controls (with BCS1L being the strongest at 0.869), none survived FDR correction. The best-performing 10-protein panel achieved only a cross-validated AUC of 0.742, significantly lower than the inflated AUC of 0.946 obtained without proper cross-validation. This underscores the risk of overfitting when features vastly outnumber samples. The fundamental limitation lies in the profound heterogeneity of autoantibody profiles. Notably, 67.5% of PANDAS-positive targets were reactive in only a single patient, and no protein was universally positive across all 13 PANDAS subjects. Critically, the targets that best discriminated PANDAS from healthy controls largely failed to discriminate PANDAS from autism spectrum disorder (ASD). Many top performers (e.g., BCS1L, VEGFA, and SIGMAR1) showed substantial AUC drops when tested against ASD, indicating that they detect autoantibody reactivity shared across neuropsychiatric conditions rather than PANDAS-specific immunity. Conversely, PANDAS versus ASD discrimination required an almost entirely distinct target set (e.g., PRH2, RBM25, and PDCD1LG2), with only nine proteins achieving an AUC ≥ 0.75 in both comparisons simultaneously. This comparison-dependence was mirrored in the panel classifiers, which performed moderately against healthy controls but at or below chance against ASD. Taken together, these findings indicate that autoantibody-based diagnosis of PANDAS is fundamentally constrained by inter-patient heterogeneity and the overlap of autoantibody reactivity with other neuropsychiatric conditions, rather than by insufficient analytical sensitivity. Subsampling analysis confirmed that the failure to discriminate PANDAS from ASD reflects both the instability of panel classifiers with small comparator groups and genuine sharing of autoantibody reactivity between the two neuropsychiatric conditions.

Our second goal was to search for targets of PANDAS-associated IgG that may be related to the disease pathogenesis. We did not observe changes in circulating levels of NfL in either PANDAS or ASD subjects compared to healthy controls, which indicated that neither condition in our patient cohort was associated with measurable neuronal damage. While this seemingly contradicts studies that demonstrated elevated NfL levels in ASD (Simone et al., 2023; Oz et al., 2024), there are inconsistent pieces of literature supporting NfL as a reliable ASD biomarker (Bavato et al., 2024; Paketci et al., 2022).

Functional enrichment analysis revealed that the 117 PANDAS-specific targets were significantly enriched for neuronal, epigenetic, and immune-regulatory functions. These functions include transcription factors, chromatin remodelers (HDAC4/5 and DNMT3A), apoptosis regulators (BCL2), and neural developmental proteins (BCL11A, LHX2, SH3GL3). Despite their individual heterogeneity, these autoantibodies converge on biologically coherent pathways that are relevant to PANDAS pathogenesis.

The predominance of intracellular targets may have important implications for the pathogenesis of PANDAS. If autoantibodies are the primary effectors of disease, the 12 antibody-accessible targets—particularly the immune checkpoint receptor LAG3 and the calcium-activated chloride channel ANO1—represent the most plausible candidates for direct autoantibody-mediated pathology. The remaining intracellular targets likely reflect secondary autoantibody responses arising through epitope spreading following initial tissue injury, wherein damage to neurons or other cells releases intracellular proteins that are subsequently recognized by the adaptive immune system. This interpretation is consistent with the observation that most intracellular targets are positive in only a single PANDAS patient, as the specific pattern of released antigens would depend on the location and extent of tissue damage in each subject. Alternatively, these autoantibodies may have been generated through molecular mimicry with streptococcal antigens that share structural homology with the identified intracellular targets.

Several individual PANDAS-specific targets merit attention for their potential roles in disease pathogenesis (Table 8). Among immune-regulatory targets, CIITA is the master transcriptional activator of MHC class II genes; autoantibodies against CIITA could impair antigen presentation and disrupt adaptive immune regulation. LAG3 is an immune checkpoint receptor whose blockade enhances T cell activation; anti-LAG3 autoantibodies could have similar activating effects, promoting autoimmune T cell responses. Among neural targets, HDAC4 and HDAC5 are class IIa histone deacetylases that shuttle between the nucleus and cytoplasm in response to neuronal activity and are essential for synaptic plasticity and learning; both are highly expressed in the basal ganglia, the brain region most implicated in PANDAS. PTPN11 (SHP2) is a tyrosine phosphatase that mediates RAS-MAPK signaling downstream of multiple growth factor receptors; germline gain-of-function mutations cause Noonan syndrome, which features neurodevelopmental abnormalities. BCL11A is a transcription factor required for normal brain development whose haploinsufficiency causes Dias-Logan syndrome with intellectual disability, and BCL2 is a master anti-apoptotic regulator whose inhibition could promote neuronal cell death. Future research will be necessary to validate and reconcile them with the data from the structure-focused work from the Pittenger lab, similar to the authors’ description of DRD2 dopamine receptor targeting in the striatal cholinergic interneurons (ChIs) (Xu et al., 2021; Xu et al., 2024; Frick et al., 2018).

**Table 8:** Key PANDAS-specific autoantibody targets with potential pathogenic relevance, organized by functional category.

Gene	Category	Pathogenesis relevance
CIITA	Immune	Master regulator of MHC class II; autoantibodies could impair antigen presentation and adaptive immunity
LAG3	Immune/Surface	T cell immune checkpoint receptor; anti-LAG3 autoantibodies may disrupt T cell homeostasis
PTPN11	Signaling	SHP2 phosphatase in RAS-MAPK pathway; germline mutations cause Noonan syndrome (neurodevelopmental)
HDAC4	Epigenetic/Neural	Class IIa HDAC critical for synaptic plasticity; mutations cause brachydactyly-intellectual disability
HDAC5	Epigenetic/Neural	Class IIa HDAC regulating neuronal gene expression; enriched in basal ganglia
BCL11A	Neural/Apoptosis	Essential for brain development; haploinsufficiency causes Dias-Logan syndrome (intellectual disability)
DNMT3A	Epigenetic	De novo DNA methyltransferase; mutations cause Tatton-Brown-Rahman syndrome (overgrowth + ID)
BCL2	Apoptosis	Anti-apoptotic; autoantibodies could promote neuronal cell death
SH3GL3	Synaptic	Endophilin A3; synaptic vesicle recycling at nerve terminals
TCF7L2	Signaling	Wnt pathway effector; implicated in neuropsychiatric conditions
ANO1	Surface/Neural	Calcium-activated chloride channel (TMEM16A); expressed in neurons and smooth muscle
PRL	Immune/Secreted	Prolactin; immunomodulatory hormone that promotes autoimmunity at elevated levels

Assignments were based on UniProt and Human Protein Atlas annotations.

We found that (77.8%) of protein targets specific to PANDAS were enriched for disordered regions, which are among the strongest predictors of linear B cell epitope immunogenicity. Those highly immunogenic proteins are frequently released during cell damage in a partially denatured state, and their enrichment is consistent with the epitope spreading model: streptococcal infection → initial tissue damage → release of intracellular IDR-rich proteins (transcription factors, chromatin remodelers, signaling molecules) → secondary autoantibody responses against these exposed disordered domains.

One PANDAS patient (PS31) received intravenous immunoglobulin (IVIG) therapy around the time of serum collection, raising the concern that exogenous polyclonal antibodies could have introduced reactivities unrelated to the patient’s endogenous autoimmune response. To address this potential confounder, we performed a sensitivity analysis in which PS31 was excluded, and the entire downstream pipeline was re-run on the remaining 12 PANDAS samples. PS31 contributed to only 4 of the 117 PANDAS-specific targets (3.4%), of which 3 were singletons unique to PS31 (PPP5D1, ANO1, and CENPT) and one (PLEKHM3) was also positive in two other PANDAS patients. Exclusion of PS31 yielded 114 PANDAS-specific targets, preserved the patient-specific heterogeneity (90.4% singletons vs. 90.6% in the original analysis), and left every functional category enrichment intact, with fold-enrichment values and statistical significance marginally strengthened rather than diminished (Supplementary Tables S1–S3). The minimal impact of PS31 exclusion argues against substantial IVIG-related contamination of the autoantibody profile and supports the robustness of the reported findings.

There are several limitations of our study. Our patient groups were relatively small. PANDAS, classified as a rare disease by the Genetic and Rare Diseases Information Center (GARD:7312) and Orphanet (ORPHA:66624), has a low prevalence. This limited availability of suitable, well-characterized subjects for research contributes to our small sample size. Additionally, the absence of a specific ICD-10 code for PANDAS, often relying on proxies like D89.89 (other specified immune disorders), complicates epidemiological tracking and case identification in clinical databases. These challenges further hinder the assembly of larger cohorts. It is plausible that our modest cohort size may have affected our ability to detect targets with low autoantibody titers. Also, females were over-represented in the PANDAS group, which appears to contrast with a clinical study with Italian children, which reported a male/female ratio of 3.33:1 in a small cohort of 26 children with PANDAS (Leonardi et al., 2023). No additional published epidemiological data confirmed this distribution so far, and statistical comparison of immunoreactivity of 15,006 targets in the initial screen showed that sex was not a statistically significant confounding variable. Thus, the skewed representation of males and females in our small cohort was unlikely to affect the analysis outcomes. However, considering the small size of our PANDAS cohort (*n* = 3 for males), we cannot exclude the possibility that in a larger study of this kind, sex could have a more considerable effect on the measured variables.

Finally, while the NAPPA protein arrays allow for an unbiased screening of autoantibodies directed against full-length human proteins, the associated method of *in vitro* transcription/translation allows for the detection of autoantibodies directed toward linear epitopes only, but may not allow for proper protein folding (lack of accessory chaperone proteins) or post-translational modifications, which may determine antibody reactivity toward conformational or neo-epitopes. Complementary high-throughput approaches such as PhIP-Seq, which screens autoantibody reactivity against phage-displayed peptide libraries spanning the human proteome, could provide independent validation and peptide-level epitope mapping of the autoantibody targets identified here. However, like NAPPA, PhIP-Seq is limited to linear epitope detection and would not address the conformational epitope limitation.

## Conclusion

Despite the shortcomings, our evaluation of autoantibody targets in PANDAS provides an original and novel insight into the heterogeneity of the humoral response among patients and highlights the potential limitations in designing a reliable serological diagnostic tool. Our dataset may provide an important platform for further research into autoantigens of potential pathogenic importance. Further investigations with a larger number of subjects/samples and alternative techniques are needed.

## Data Availability

The original contributions presented in the study are included in the article/Supplementary material, further inquiries can be directed to the corresponding author.

## Footnotes

1
DNASU.org

2
https://davidbioinformatics.nih.gov/

3
string-db.org
