# Supplementary material for: Unbiased autoantibody screening using nucleic acid protein programmable array in pediatric autoimmune neuropsychiatric disorder associated with streptococcal infections
Source: Front Behav Neurosci. 2026 Apr 29;20:1774848. doi: 10.3389/fnbeh.2026.1774848 (PMC13168177; doi:10.3389/fnbeh.2026.1774848)
Supplement: Supplementary file 1 [file Data_Sheet_1.pdf]

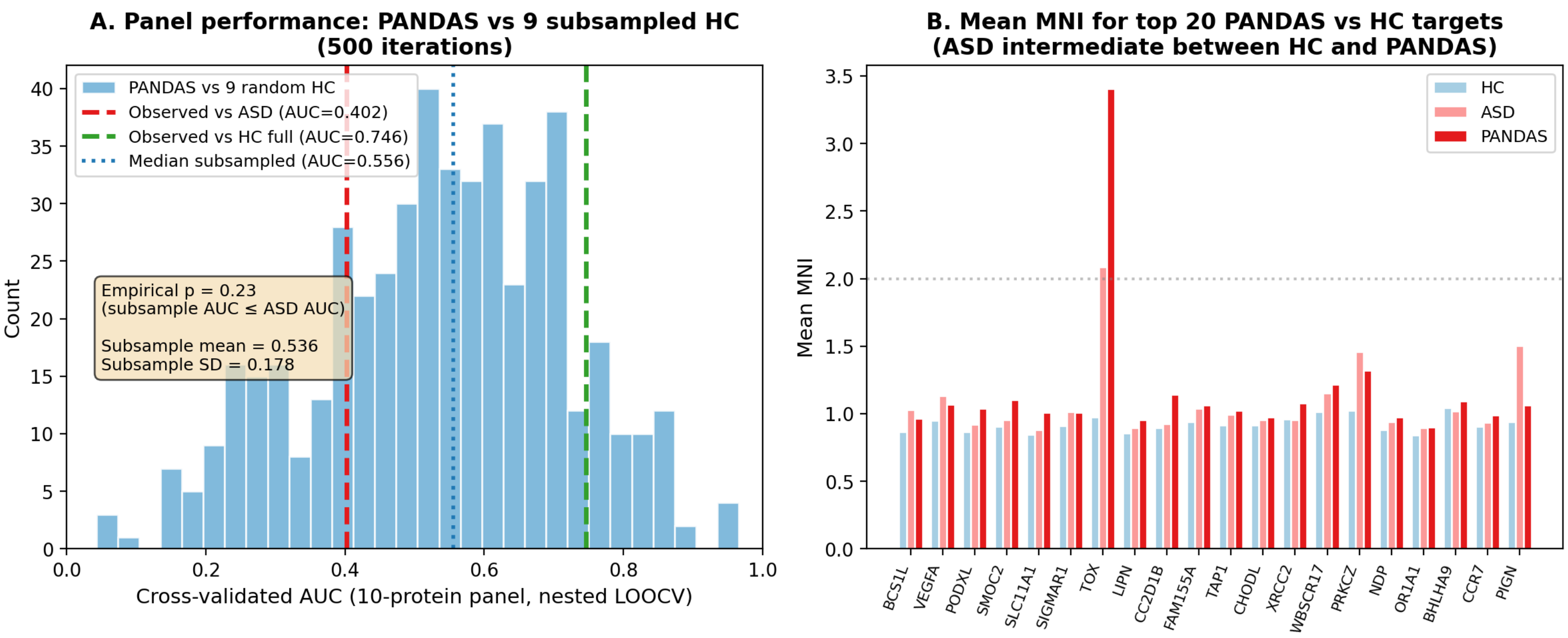

Supplemental Figure S1. Subsampling analysis to distinguish power limitation from biological overlap in PANDAS vs ASD classification failure. (A) Distribution of cross-validated AUC values from 10-protein panel classifiers (L2-regularized logistic regression, nested LOOCV) trained on PANDAS (n=13) vs 9 randomly subsampled healthy controls (500 iterations). The median subsampled AUC (0.556, blue dotted line) was substantially lower than the full HC comparison (0.746, green dashed line), demonstrating classifier instability when the comparator group is reduced to n=9. The observed PANDAS vs ASD AUC (0.402, red dashed line) fell within the subsampled distribution (empirical p=0.23), indicating that reduced sample size partially accounts for the poor ASD discrimination. **(B)** Mean Median Normalized Intensity (MNI) for the top 20 individual PANDAS vs HC discriminators across all three groups. For 18 of 20 targets, ASD subjects (pink) showed mean MNI values intermediate between or exceeding those of PANDAS (red), rather than resembling HC (blue), indicating that autoantibody reactivity at these targets is shared between the two neuropsychiatric conditions. Dashed gray line indicates the MNI=2 positivity threshold. Together, these analyses indicate that the failure of panel classifiers to discriminate PANDAS from ASD reflects both limited statistical power with 9 ASD subjects and genuine biological overlap in autoantibody profiles between PANDAS and ASD.
