# Supplementary material for: Unbiased autoantibody screening using nucleic acid protein programmable array in pediatric autoimmune neuropsychiatric disorder associated with streptococcal infections
Source: Front Behav Neurosci. 2026 Apr 29;20:1774848. doi: 10.3389/fnbeh.2026.1774848 (PMC13168177; doi:10.3389/fnbeh.2026.1774848)
Supplement: Supplementary file 2 [file Data_Sheet_2.pdf]

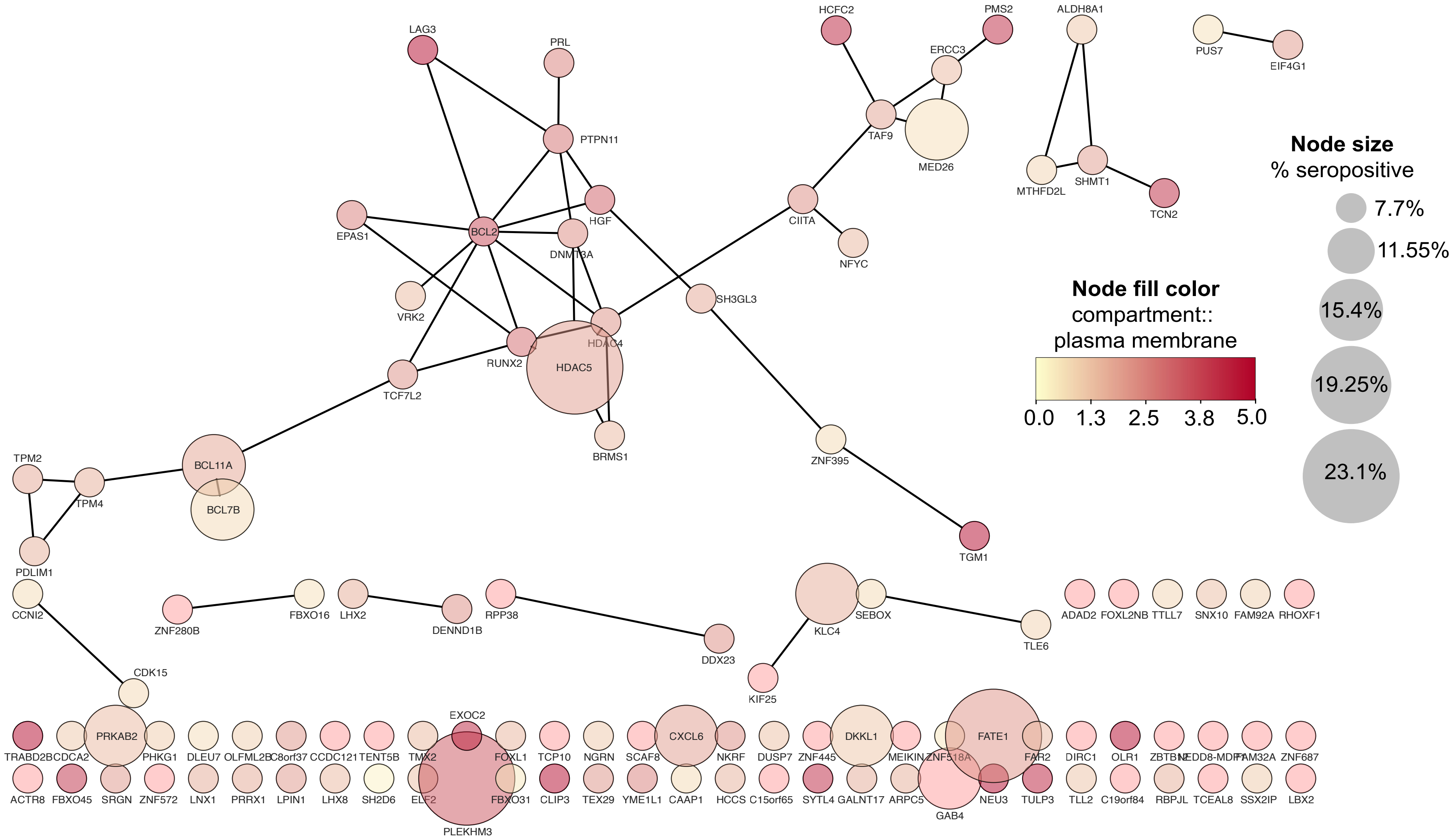

Supplemental Fig. S2: Nodes represent PANDAS-specific autoantibody protein targets (n = 115). Node color indicates subcellular localization confidence based on the COMPARTMENTS database (Binder et al., 2014), scored on a 0–5 scale integrating curated knowledge, experimental evidence, text mining, and sequence predictions. Node size is proportional to the percentage of PANDAS patients seropositive for each target. Edges represent known or predicted protein-protein interactions from STRING (confidence ≥ 0.4).
