# Supplementary material for: Unbiased autoantibody screening using nucleic acid protein programmable array in pediatric autoimmune neuropsychiatric disorder associated with streptococcal infections
Source: Front Behav Neurosci. 2026 Apr 29;20:1774848. doi: 10.3389/fnbeh.2026.1774848 (PMC13168177; doi:10.3389/fnbeh.2026.1774848)
Supplement: Supplementary file 3 [file Data_Sheet_3.pdf]

**Table S1. Summary comparison of PANDAS-specific targets with and without PS31.**

| Metric | Original (n = 13) | Sensitivity (n = 12, PS31 excluded) | Change |
|---|---|---|---|
| Total PANDAS-specific targets | 117 | 114 | −3 (−2.6%) |
| Targets positive in 1 patient only | 106 (90.6%) | 103 (90.4%) | −3 |
| Targets positive in 2 patients | 8 | 9 | +1 |
| Targets positive in 3 patients | 3 | 2 | −1 |
| Targets shared by ≥2 patients | 11 (9.4%) | 11 (9.6%) | 0 |

**Table S2. Detailed characterization of PS31-positive PANDAS-specific targets.**

| Gene | PS31 MNI | Other PANDAS patients positive | Status after PS31 exclusion |
|---|---|---|---|
| **PLEKHM3** | 3.24 | PS9, PS28 | Retained (still positive in 2 patients) |
| **PPP5D1** | 2.15 | (none) | Lost (PS31-only singleton) |
| **ANO1** | 2.24 | (none) | Lost (PS31-only singleton) |
| **CENPT** | 2.77 | (none) | Lost (PS31-only singleton) |

**Table S3. Functional category enrichment comparison.**

| Category | k/K | Fold | p-value | k/K | Fold | p-value |
|---|---|---|---|---|---|---|
| | Original (n=13) | Sensitivity (n=12) | | | | |
| Transcription factor | 20/20 | 3.22 | 9.68e−10 | 20/20 | 3.30 | 3.89e−22 |
| Apoptosis (cell death regs) | 5/5 | 10.56 | 7.03e−06 | 4/4 | 10.84 | 6.90e−05 |
| Neural development / function | 9/19 | 5.00 | 1.86e−05 | 9/19 | 5.13 | 1.31e−05 |
| Chromatin / epigenetic | 5/9 | 5.87 | 6.47e−04 | 5/9 | 6.02 | 5.60e−04 |
| F-box / ubiquitin-proteasome | 5/9 | 5.87 | 6.47e−04 | 5/9 | 6.02 | 5.60e−04 |
| Cytoskeleton / motility | 8/8 | 10.56 | 5.17e−09 | 8/8 | 10.84 | 4.16e−09 |
| Immune regulation | 7/7 | 10.56 | 5.78e−08 | 7/7 | 10.84 | 4.78e−08 |
| Signal transduction | 6/6 | 10.56 | 6.40e−07 | 6/6 | 10.84 | 5.45e−07 |
| Vesicle trafficking | 6/6 | 10.56 | 6.40e−07 | 6/6 | 10.84 | 5.45e−07 |
